# Body mass index, body dissatisfaction and adolescent smoking initiation

**DOI:** 10.1016/j.drugalcdep.2017.04.008

**Published:** 2017-09-01

**Authors:** Laurence J. Howe, Lea Trela-Larsen, Michelle Taylor, Jon Heron, Marcus R. Munafò, Amy E. Taylor

**Affiliations:** a MRC Integrative Epidemiology Unit, University of Bristol, Bristol, BS8 2BN, UK; b Musculoskeletal Research Unit, University of Bristol, Learning and Research Building (Level 1), Southmead Hospital, Bristol, BS10 5NB, UK; c School of Social and Community Medicine, University of Bristol, Bristol, UK; d UK Centre for Tobacco and Alcohol Studies, School of Experimental Psychology, University of Bristol, Bristol, BS8 1TU, United Kingdom

**Keywords:** Tobacco, Body mass index, Body dissatisfaction, Mendelian randomization, ALSPAC

## Abstract

**Background:**

Smoking influences body weight, but there is little evidence as to whether body mass index (BMI) and body dissatisfaction increase smoking initiation in adolescents.

**Methods:**

We evaluated the association between measured BMI, body dissatisfaction and latent classes of smoking initiation (never smokers, experimenters, late onset regular smokers, early onset regular smokers) in the Avon Longitudinal Study of Parents and Children. In observational analyses we used BMI (N = 3754) and body dissatisfaction at age 10.5 years (N = 3349). In Mendelian randomisation (MR) analysis, we used a BMI genetic risk score of 76 single nucleotide polymorphisms (N = 4017).

**Results:**

In females, higher BMI was associated with increased odds of early onset regular smoking (OR: 1.11, 95% CI: 1.04, 1.18) compared to being a never smoker, but not clearly associated with experimenting with smoking (OR: 1.04, 95% CI: 0.99, 1.10) or late onset regular smoking (OR: 1.01, 95% CI: 0.94, 1.09). No clear evidence was found for associations between BMI and smoking initiation classes in males (*p*-value for sex interaction ≤ 0.001). Body dissatisfaction was associated with increased odds of late-onset regular smoking (OR: 1.71, 95% CI: 1.32, 1.99) in males and females combined (P-value for sex interaction = 0.32). There was no clear evidence for an association between the BMI genetic risk score and smoking latent classes in males or females but estimates were imprecise.

**Conclusions:**

BMI in females and body dissatisfaction in males and females are associated with increased odds of smoking initiation, highlighting these as potentially important factors for consideration in smoking prevention strategies.

## Highlights

• Smoking has been shown to affect body weight but evidence for the converse is limited.
• Higher body mass index (BMI) was associated with smoking initiation in adolescent females but not males.
• Body dissatisfaction was associated with smoking initiation in both sexes.
• BMI genetic risk score did not predict smoking, but estimates were imprecise.
• BMI and body dissatisfaction may be important considerations for smoking prevention.

## 1 Introduction

Recent figures suggest that 207,000 children between the ages of 11 and 15 start smoking each year in the United Kingdom, with around 80% of adult smokers taking up smoking before the age of 20 (ASH, 2015). Therefore, preventing uptake of smoking in adolescence is of paramount importance. Smoking is associated with lower body weight (Chiolero et al., 2008) and there is good evidence that this link is causal (Freathy et al., 2011). However, the causal effect of body weight on smoking is largely unknown. There is some evidence that high body mass index (BMI) is a possible risk factor for smoking initiation because people may start smoking in order to control or lose weight. In one study, adolescents who reported trying to lose weight had increased rates of smoking initiation (Strauss and Mir, 2001). In another study, adolescent female smokers were no more likely to be trying to lose weight than non-smokers (Nichter, 2004). However, the majority of studies investigating links between body weight and smoking are cross-sectional and therefore might be subject to reverse causation.

Body dissatisfaction could be a mediator or confounder of the relationship between BMI and smoking; high BMI may cause body dissatisfaction which then leads to increased smoking behaviour (mediator) or, alternatively, body dissatisfaction could independently lead to both changes in BMI and smoking behaviour (confounder). In general, observational studies have found body dissatisfaction or weight concerns are risk factors for smoking in females but not males (Cawley et al., 2004, French et al., 1994, Tomeo et al., 1999, Winter et al., 2002). A review of studies investigating body weight concerns and tobacco use concluded that the evidence for a positive association depends largely on the dimension of the weight concern variable considered (e.g., dietary behaviour, disordered eating), and that the positive association between body dissatisfaction and smoking was more consistent amongst female adolescents than males (Potter et al., 2004). Unravelling the complex relationships between BMI, body dissatisfaction and adolescent smoking behaviour is of clinical importance as it may allow the identification of adolescents at greater risk of tobacco smoking and allow interventions to be targeted appropriately.

**Fig. 1:**
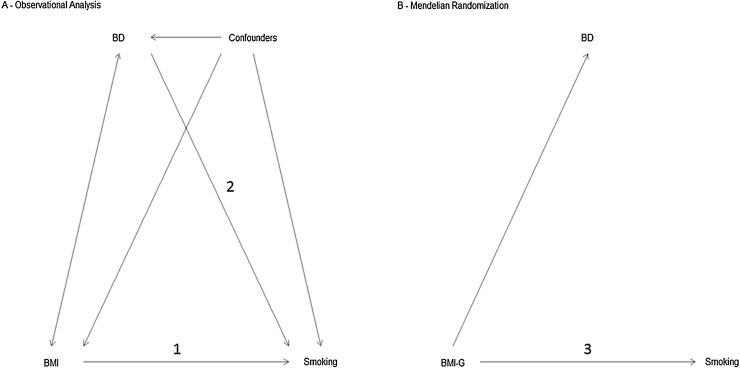
Diagrams of the primary analyses. These diagrams show the pathways being tested in the analysis only. There is evidence from the literature that the smoking and body mass index (BMI) relationship is bi-directional, however we have not shown this in the figure. Figure A shows the observational analysis conducted, which examined (1) The relationship between BMI at age 10.5 years and patterns of smoking between the ages of 13 and 18 years and (2) the relationship between body dissatisfaction (BD) at age 10.5 years and patterns of smoking between the ages of 13 and 18 years. Figure B demonstrates the Mendelian randomization analysis (3) which used a genetic risk score as a proxy for BMI (BMI-G) to examine the relationship with patterns of smoking between the ages of 13 and 18 years and with body dissatisfaction.

Using data from a large longitudinal study, the Avon Longitudinal Study of Parents and Children (ALSPAC), we examined the relationship between BMI, body dissatisfaction and the smoking habits of adolescents. We used latent classes of smoking initiation described previously (Heron et al., 2011), but extended to 18 years. First, we examined the relationships between BMI and body dissatisfaction at age 10.5 years with subsequent adolescent smoking initiation between the ages of 13 and 18. Second, we used a genetic risk score as a proxy for measured BMI in a Mendelian randomisation (MR) approach. We were primarily interested in evaluating the effect of BMI and body dissatisfaction on smoking while also interested in the nature of the relationship between BMI and body dissatisfaction (Fig. 1).

MR is an instrumental variable approach that uses genetic variant(s) associated with an exposure to examine the relationship between that exposure and an outcome. In principle it should be less susceptible to problems of confounding and reverse causation that can affect observational studies (Davey-Smith and Ebrahim, 2003) and is often used as a method of causal inference. MR relies on three assumptions: firstly that the instrument is robustly associated with the exposure, secondly that the only association between the instrument and the outcome is through the exposure and thirdly that there are no common determinants of the instrument and the outcome (e.g., population stratification) (Solovieff et al., 2013). Evidence for a causal role of smoking in reducing body weight has been found using MR methods (Åsvold et al., 2014, Freathy et al., 2011). Higher genetically determined BMI (using a polygenic risk score based on 32 genetic variants) was found to be associated with smoking more cigarettes per day and increased risk of smoking initiation (Thorgeirsson et al., 2013). These results were interpreted by the authors as evidence for a shared genetic aetiology between smoking and BMI, but the results could also be interpreted as evidence for weight influencing smoking behaviour.

## 2 Material and methods

### 2.1 Study participants

We used data on children from the Avon Longitudinal Study of Parents and Children (ALSPAC), a longitudinal study that recruited pregnant women living in the former county of Avon (UK) with expected delivery dates between 1 April 1991 and 31 December 1992. Avon is a county in the southwest of the United Kingdom (UK), 120 miles west of London with a population of 1 million. Children living in Avon were surveyed and found to be broadly similar to the UK population in terms of socio-economic and ethnicity related demographics (Golding et al., 2001).

The initial number of enrolled pregnancies was 14,541, which resulted in 14,062 live births and 13,988 children alive at the age of 1. When the oldest children were approximately 7 years of age, the initial sample was boosted with eligible cases who had failed to join the study originally. Full details of the enrolment have been documented elsewhere (Boyd et al., 2013, Golding et al., 2001). Data have been gathered from the mother and her partner (during pregnancy and post birth) and the children from self-report questionnaires and clinical sessions. Ethics approval for the study was obtained from the ALSPAC Ethics and Law Committee and the Local Research Ethics Committee. The study website contains details of all available data through a searchable data dictionary (http://www.bristol.ac.uk/alspac/researchers/dataaccess/datadictionary/).

### 2.2 Measures

#### 2.2.1 Smoking behaviour

The measures of smoking behaviour used in these analyses were collected at six time points. At 14, 16 and 18 years, data were collected using self-completed postal questionnaires, while data at 13, 15 and 17 years were collected during clinic visits. For the clinics at 15 and 17 years the smoking questions were answered using a computer-based questionnaire. The number of participants with smoking frequency data ranged from 5813 at 13 years to 3209 at 18 years. Responses to one or more questions at each time point were used to derive a repeated 4 level ordinal variable with categories “Non-smoker”, “Occasional smoker” (typically less than once per week), “Weekly smoker” and “Daily smoker” (Supplementary Table 1).

#### 2.2.2 Body mass index

BMI was calculated using height (m) and weight (kg) measured at a clinic session when the children were aged 10 years (mean: 10.7 years; inter-quartile range: 10.5 years to 10.8 years).

#### 2.2.3 Body dissatisfaction

Perceived and desired body shape were assessed using Stunkard figure rating scales (Stunkard et al., 1983) in a questionnaire administered at 10.5 years (mean: 10.7 years; inter-quartile range: 10.7 years to 10.8 years). The Stunkard figures are illustrations of different body types ranging from endomorph to ectomorph and participants were asked to select their perceived and desired body shape from the illustrations. A body dissatisfaction score was generated by taking the absolute value of the difference between the perceived and desired body shapes (i.e., a higher score means greater body dissatisfaction. The score varied from 0 (no difference in perceived and desired body shapes) to 4 (perceived body shape opposite to desired). As there were only a few individuals scoring above 1 (N = 122) the score was simplified to a binary variable; no body dissatisfaction (score of 0) or body dissatisfaction (score ≥ 1).

#### 2.2.4 Covariates

Covariates included sex, mother’s highest education (“CSEs”: Certificate of Secondary Education, examinations at age 16, “Vocational”: education specific to a trade, “O Level”: examinations at age 16 aimed at more academic pupils, “A level”: examinations at age 18, “Degree”: university degree) reported by the mother at 32 weeks gestation, maternal smoking (“never smoked”, “former smoker”, “current smoker”) reported by the mother when the child was 11 years of age, parity (number of previous pregnancies) reported by the mother at 18 weeks gestation, housing tenure (“Owned”, “Mortgaged”, “Private Rented”, “Council Rented”, “Other”) reported by the mother at 8 weeks gestation, crowding status (number of co-residents/number of rooms split into 4 categories “≤0.5”, “0.5–0.75”, “0.75–1” and “1<”) reported by the mother at 8 weeks gestation, a total behavioural score at age 11 (a summation of variables related to emotional symptoms, conduct problems, hyperactivity score and peer relationship problems) from the Strengths and Difficulties Questionnaire (SDQ) (Goodman et al., 2000) and the age of BMI clinic measurement (weeks).

### 2.3 Genotyping

A total of 9912 ALSPAC children were genotyped using the Illumina HumanHap550 quad genome-wide SNP genotyping platform. The genotyped sample, consisted of unrelated individuals of European ancestry. More information on quality control is provided in Supplementary Methods. Polygenic risk scores for BMI were constructed using 77 SNPs previously found to be associated with BMI at genome-wide significance level in European adults (Locke et al., 2015). Of these 76 were directly genotyped or imputed in ALSPAC and met quality control criteria (Supplementary Table 2). We calculated weighted risk scores, using the effect sizes (beta coefficients) from the GIANT study (Locke et al., 2015). Weighted risk scores were standardised (converted to Z scores). As one of the BMI related SNPs (rs11030104 in *BDNF*) is associated with smoking initiation at a genome-wide significance level (Tobacco and Genetics Consortium, 2010), we also created a risk score excluding this SNP.

### 2.4 Statistical analysis

Longitudinal latent class analysis (LLCA) was used to extract patterns of smoking behaviour. Participants with extensive missing data would be difficult to assign to classes with any confidence; therefore, only study participants with data at 3 or more of the time points (3+ measures) were included. To establish the optimal number of latent classes, we used: (a) the Bayesian Information Criterion (BIC) (Schwarz, 1978); (b) the Bootstrap Likelihood Ratio Test (BLRT) (Nylund et al., 2007); (c) the Vuong-Lo-Mendell Likelihood Ratio Test (VLMR) (Lo et al., 2001); (d) the Lo-Mendell Likelihood Ratio Test (LMR) (Lo et al., 2001); and (e) entropy (Jedidi et al., 1993). The latent class models were re-parametrised so that comparisons between classes could be attained. The “Modal ML” three-step method proposed by Vermunt (Vermunt, 2010) was used to obtain parameter estimates that enable the inclusion of outcome data without distorting the latent class solution. In the first step, the latent class model is estimated using an unconditional LLCA model (i.e., model with no covariates) and this model is then used to derive class-assignment probabilities (i.e., the probability that each individual belongs to each class). In step two, individuals are assigned to the class for which their probability is the greatest creating a non-latent classification. Finally in the third step, the measurement error in the non-latent classification is quantified and used to reproduce latent classes that use a set of logit constraints. This method has been shown to provide less-biased estimates than the standard three-step methods whilst avoiding the effect of covariates on the measurement model, an issue in one-step models (Heron et al., 2015).

Associations between BMI and body dissatisfaction and membership of the smoking latent classes were estimated using a series of univariate multinomial logistic regression models using the most populous class as the baseline category. First, we ran an observational model examining the association between measured BMI and smoking latent classes with adjustment for the covariates described above. Second, we ran an observational model examining the association between body dissatisfaction and smoking latent classes with adjustment for all covariates described above. Third, we ran an MR model examining the association between BMI genetic risk scores and smoking latent classes. This was adjusted for sex. As it was hypothesised that the relationship between BMI/body dissatisfaction and smoking would be different in males and females, we stratified the analyses by gender and tested for an interaction. Fig. 1 illustrates the differences between the three models and Supplementary Fig. 1 shows how the final study samples were reached. Analyses were conducted in MPlus 7.31 (Muthén and Muthén, 2005) and Stata (version 13.1) (Stata, 2013).

## 3 Results

### 3.1 Latent classes

Between ages 13–18 years, 1497 participants had complete information on smoking frequency available. The latent class analysis was based on 5335 participants who had information on smoking frequency available for at least three of the available time points.

**Fig. 2:**
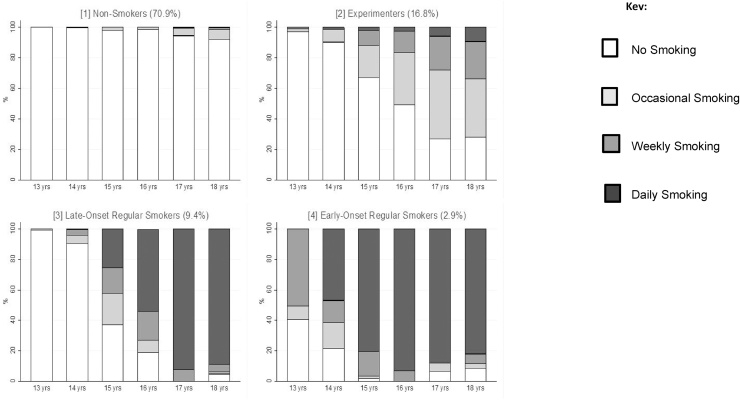
Distribution of smoking responses across latent classes at each time point for smoking data-set (N = 5335). Class proportions shown as % based on estimated posterior probability.

A 4-class model was selected for the smoking trajectory data over a lower or higher number of classes. Details regarding the model fit statistics and a discussion of our decision process can be found in Supplementary Table 3. This 4-class solution comprised smoking behaviour patterns that we refer to as: never-smokers (70.9%), experimenters (16.8%), late-onset regular smokers (9.4%) and early-onset regular smokers (2.9%) (Fig. 2). In general never-smokers did not report smoking across the 6 time-points, the experimenter’s class was characterised by occasional or weekly smoking in some of the later time points, and the late-onset regular smoking class was characterised by daily smoking by the age of 17, while the early-onset regular smoking class was characterised by daily smoking by 15 or 16 years.

### 3.2 Body mass index, body dissatisfaction and smoking

There were more females than males in both the observational (53.9%) and MR (56.0%) samples. The mean BMI was 17.9 kg/m^2^ (range: 12.9–33.2) in males and 18.2 kg/m^2^ (range: 12.4–33.3) in females, in the observational sample. The mean BMI was 17.9 kg/m^2^ (range: 12.8–33.2) in males and 18.2 kg/m^2^ (range: 12.4–36.1) in females in the MR sample (Supplementary Table 4). The overlap between the observational and MR samples was 3511 individuals comprising 92.7% of the observational sample and 87.4% of the MR sample. Mother’s education was found to be associated with BMI in both males and females while maternal smoking and housing tenure were associated with BMI in females only (Supplementary Table 5)

#### 3.2.1 Body mass index

There was strong statistical evidence for an interaction between sex and BMI in the association with smoking latent classes (P < 0.001) (Table 1). For males, there was no clear evidence that higher BMI was associated with any of the smoking behaviour classes compared to never-smoking. For females, higher BMI was associated with increased odds of becoming a smoker in early adolescence compared to being a never-smoker but there was no clear evidence that higher BMI was associated with increased odds of experimenting with smoking or becoming a late-onset smoker (Table 1).

**Table 1:** The formatting of Tables 1 and 2 in the pdf copy have gaps in between the unadjusted and partially adjusted rows making it difficult to read. The formatting is fine in the proof copy. Association between unit increase in measured body mass index at age 10 and classes of smoking initiation (odds ratios).

		Class (percentage membership)				
		Never (71.0%)	Experimenters (16.6%)	Late Onset (9.1%)	Early Onset (3.3%)	P Value^d^
Male (n = 1732)	Unadjusted^a^	1.00 (REF)	0.96 (0.89, 1.03)	1.04 (0.99, 1.10)	1.00 (0.90, 1.10)	0.23
	Partially adjusted^b^	1.00 (REF)	0.95 (0.88, 1.02)	1.03 (0.97, 1.08)	0.98 (0.88, 1.08)	0.30
	Fully adjusted^c^	1.00 (REF)	0.93 (0.84, 1.02)	0.99 (0.91, 1.06)	1.04 (0.95, 1.13)	0.34
Female (n = 2022)	Unadjusted^a^	1.00 (REF)	1.04 (0.99, 1.08)	1.11 (1.05, 1.17)	1.14 (1.07, 1.21)	<0.001
	Partially adjusted^b^	1.00 (REF)	1.04 (0.99, 1.09)	1.10 (1.04, 1.15)	1.13 (1.06, 1.20)	<0.001
	Fully adjusted^c^	1.00 (REF)	1.04 (0.99, 1.10)	1.01 (0.94, 1.09)	1.11 (1.04, 1.18)	<0.001

a Adjusted for age of body mass index measurement and sex.

b Additionally adjusted for parity, mother’s education, maternal smoking, housing tenure, crowding status and total behavioural score on the Strengths and Difficulties questionnaire.

c Additionally adjusted for body dissatisfaction (sample size reduced to 1549 males and 1825 females).

d P values on inclusion of covariate to the model from Wald Test of parameter constraints.

#### 3.2.2 Body dissatisfaction

There was strong evidence that body dissatisfaction at age 10.5 years was associated with the smoking latent classes after adjustment for potential confounders including BMI (P = 0.004). There was no strong evidence for an interaction between sex and body perception in the full model (P = 0.32) (Table 2). In males and females combined, after adjustment for covariates and BMI, body dissatisfaction was associated with increased odds of becoming a late-onset smoker compared to being a never-smoker. Although the associations between body dissatisfaction and experimental smoking and between body dissatisfaction and early-onset smoking were positive, statistical evidence for these associations was weaker. Observationally a one-unit increase in BMI was associated with increased odds of body dissatisfaction in both males (OR: 1.23, 95% CI 1.18–1.28) and females (OR: 1.31, 95% CI 1.26–1.36) (Supplementary Table 5).

**Table 2:** Association between body dissatisfaction score (≥1 vs 0) and classes of smoking initiation (odds ratios).

		Class (percentage membership)				
		Never (70.1%)	Experimenters (16.3%)	Late Onset (8.5%)	Early Onset (5.2%)	P Value^d^
Males (n = 1540)	Unadjusted^a^	1.00 (REF)	1.20 (0.68, 1.71)	2.19 (1.74, 2.64)	1.19 (0.68, 1.71)	0.004
	Adjusted^b^	1.00 (REF)	1.20 (0.64, 1.75)	2.11 (1.63, 2.59)	1.09 (0.38, 1.80)	0.016
	BMI Adjusted^c^	1.00 (REF)	1.18 (0.61, 1.75)	2.14 (1.66, 2.63)	0.88 (0.12, 1.64)	0.018
Females (n = 1809)	Unadjusted^a^	1.00 (REF)	1.15 (0.80, 1.50)	1.54 (1.08, 1.99)	2.38 (1.93, 2.83)	<0.001
	Adjusted^b^	1.00 (REF)	1.13 (0.78, 1.49)	1.40 (0.93, 1.86)	2.15 (1.69, 2.62)	0.004
	BMI Adjusted^c^	1.00 (REF)	1.11 (0.74, 1.48)	1.39 (0.92, 1.87)	1.74 (1.24, 2.23)	0.069
Total (n = 3349)	Unadjusted^a^	1.00 (REF)	1.16 (0.87, 1.45)	1.82 (1.50, 2.15)	1.97 (1.61, 2.33)	<0.001
	Adjusted^b^	1.00 (REF)	1.15 (0.85, 1.45)	1.69 (1.36, 2.02)	1.73 (1.36, 2.11)	<0.001
	BMI Adjusted^c^	1.00 (REF)	1.13 (0.81, 1.45)	1.71 (1.32, 1.99)	1.41 (0.99, 1.83)	0.004

a Adjusted for age of body dissatisfaction measurement and sex.

b Additionally adjusted for parity, mother’s education, maternal smoking, housing tenure, crowding status and total behavioural score on the Strengths and Difficulties Questionnaire.

c Additionally adjusted for body mass index.

d P values on inclusion of covariate to the model from Wald Test of parameter constraints.

### 3.3 Mendelian randomisation analysis

There was strong evidence that the genetic risk score was associated with BMI: a one standard deviation increase in BMI genetic risk score was associated with a 0.74 kg/m^2^ increase in BMI (95% CI 0.57–0.90) in males, and a 0.65 kg/m^2^ increase in BMI (95% CI 0.51–0.79) in females (Supplementary Table 6). The proportion of variance in BMI explained by the genetic risk score (R^2^ coefficient) was 4.06%. In addition the BMI genetic risk score was not strongly associated with any of the confounders used in the observational analysis for either sex (Supplementary Table 6).

In the MR analysis, there was no strong evidence for an association between BMI genetic risk scores and experimental smoking, late-onset smoking or early-onset smoking, compared to never-smoking (Table 3). No clear statistical evidence was found for an interaction between sex and BMI genetic risk score (P = 0.46) on smoking initiation. Removal of the *BDNF* variant from the polygenic risk score did not alter these results substantially (Supplementary Table 7).

**Table 3:** Association between body mass index genetic risk score and classes of smoking initiation (odds ratios).

	Class (percentage membership)				
	Never	Experimenters	Late Onset	Early Onset	P value
Males (n = 1768)	1.00 (REF)	0.90 (0.70, 1.11)	0.94 (0.75, 1.12)	1.00 (0.62, 1.37)	0.69
Females (n = 2249)	1.00 (REF)	1.09 (0.94, 1.23)	0.96 (0.74, 1.18)	1.21 (0.93, 1.48)	0.45
Total (n = 4017)	1.00 (REF)	1.02 (0.90, 1.14)	0.95 (0.80, 1.09)	1.14 (0.92, 1.36)	0.66

Odds ratios are per 1 standard deviation increase in body mass index genetic risk score.

Each one standard deviation increase in BMI genetic risk score was associated with 9% increased odds of body dissatisfaction (OR 1.09, 95% CI 1.01–1.18). Odds ratios were similar for males and females. There was no strong statistical evidence of an interaction between sex and BMI genetic risk score on body dissatisfaction (P = 0.73).

## 4 Discussion

We found evidence that both BMI and body dissatisfaction in childhood are associated with smoking uptake in adolescence. Observationally, higher BMI in females was associated with increased odds of early-onset regular smoking but not increased odds of experimental smoking or late-onset smoking. Conversely in males, BMI was not associated with increased smoking uptake across any of the classes. Having high body dissatisfaction was associated with increased odds of becoming late onset regular smokers.

The positive associations between the BMI polygenic risk score and body dissatisfaction suggests that BMI may be a causal risk factor for body dissatisfaction. This is consistent with previous findings that high BMI is associated with increased body dissatisfaction in males and above average BMI is associated with increased body dissatisfaction in females (Calzo et al., 2012). However, another possible interpretation is that BMI and body dissatisfaction have a shared underlying genetic aetiology. Body dissatisfaction was associated with increased smoking initiation in both males and females, even after adjustment for BMI, suggesting body perception may be associated with smoking initiation independently of BMI. This is consistent with previous findings that body dissatisfaction is not completely driven by BMI (Micali et al., 2015). Previous literature suggests a relationship between increased body dissatisfaction and smoking, particularly for females (Cawley et al., 2004, French et al., 1994, Winter et al., 2002) but there is also evidence for this relationship existing in males (Potter et al., 2004, Tomeo et al., 1999). These studies largely agree with our finding that body dissatisfaction is strongly associated with smoking initiation in both males and females. It is difficult to determine the role of body dissatisfaction in the relationship between BMI and smoking initiation; body dissatisfaction could be a mediator or a confounder. A formal mediation analysis requires strong causal anchors and therefore strong assumptions (e.g., no unobserved confounding between BMI and body dissatisfaction as well as no measurement error). In this instance, there are many possible confounders, including depression and eating disorders, meaning that the results of a formal mediation analysis would be difficult to interpret.

Our MR analysis did not replicate the evidence found using the observational data of an association between BMI and smoking. However, the confidence intervals were wide and consistent with our observational results (Supplementary Table 8), therefore we are unable to rule out causal effects of BMI on smoking initiation from this analysis. The amount of variation in BMI at 10.5 years explained by the BMI genetic risk score was 4.06%, therefore it is possible that we did not have sufficient power to detect associations between BMI and smoking initiation using MR in this sample. Similar studies such as the study by Thorgeirsson and colleagues used sample sizes of over 100,000 and found evidence of an association between a BMI genetic risk score and both smoking initiation and cigarettes smoked per day (Thorgeirsson et al., 2013), although the smoking initiation phenotype was crude in comparison to that employed here. Future studies attempting similar MR methods should consider combining data from multiple cohorts with prospective measures of smoking initiation.

The prospective and longitudinal nature of ALSPAC is a considerable strength as it reduces the possibility of reverse causation in the associations between BMI, body dissatisfaction and smoking. Another major strength of the study is the use of multiple exposures (BMI, BMI polygenic risk score and body dissatisfaction), both observational and genetic, in the investigation of the complex body-weight smoking relationship. Nevertheless there are a number of limitations that need to be considered. First, it is difficult to identify all possible confounders and to fully account for them. Therefore the associations between BMI and body dissatisfaction and smoking presented here may still be subject to residual confounding. Second, many study participants were lost to follow-up; the initial ALSPAC sample included over 14,000 pregnancies and smoking behaviour data was collected only in 5335 participants. Loss to follow-up was more common amongst participants from less affluent families (Boyd et al., 2013) who may be more likely to take up smoking in adolescence (Heron et al., 2011). Listwise deletion remains the recommended method when dealing with missing covariates in latent class analysis, since alternative methods have clear disadvantages (Sterba, 2014b). However, there is a lack of methodological research in this area (Colder et al., 2001, Costello et al., 2008, Sterba, 2014a). Third, considering statistical power, the body dissatisfaction variable was simplified to a binary body satisfaction or body dissatisfaction variable which resulted in a loss of information. Fourth, although there is clear evidence that BMI is associated with body perception, there is evidence that both being overweight (Schwartz and Brownell, 2004) and being underweight (Watkins et al., 2008) are risk factors for body dissatisfaction in males, which suggests a non-linear relationship between BMI and body dissatisfaction. With body dissatisfaction possibly involved in the relationship between BMI and smoking initiation, BMI and smoking may also have a non-linear relationship.

The findings of our study suggest that higher BMI and body dissatisfaction are possible risk factors for subsequent adolescent smoking behaviour, especially in females. Further research is required on the relationship between body dissatisfaction and smoking in adolescence; tackling body dissatisfaction in adolescence could be important for preventing uptake of smoking.

## Conflict of interest

Marcus Munafò reports grants from Pfizer and personal fees from GlaxoSmithKline outside the submitted work. Amy Taylor reports grants from Pfizer outside the submitted work. Otherwise none.

## Contributors

Laurence Howe and Lea Trela Larsen performed the statistical analysis. Jon Heron, Michelle Taylor, Marcus Munafo and Amy Taylor formulated the research project. All authors contributed to writing of the manuscript.

## Role of funding source

Nothing declared.
