# Supplementary material for: Body mass index, body dissatisfaction and adolescent smoking initiation
Source: Drug Alcohol Depend. 2017 Sep 1;178:143–9. doi: 10.1016/j.drugalcdep.2017.04.008 (PMC5558147; doi:10.1016/j.drugalcdep.2017.04.008)
Supplement: Supplementary file 1 [file mmc1.docx]

**Supplementary Material for the Article:**

Body mass index, body dissatisfaction and adolescent smoking initiation

Laurence J. Howe ^1,3^, Lea Trela-Larsen ^2^, Michelle Taylor ^1,3^, Jon Heron ^3^, Marcus R. Munafò ^1,4^, Amy E. Taylor ^1,4^

**^1^** MRC Integrative Epidemiology Unit, University of Bristol, Bristol, BS8 2BN, UK

**^2^** Musculoskeletal Research Unit, University of Bristol, Learning and Research Building (Level 1), Southmead Hospital, Bristol, BS10 5NB, UK

**^3^** School of Social and Community Medicine, University of Bristol, Bristol

**^4^** UK Centre for Tobacco and Alcohol Studies, School of Experimental Psychology, University of Bristol, Bristol, BS8 1TU, United Kingdom

**Correspondence:**

Laurence J Howe

MRC Integrative Epidemiology Unit at the **University of Bristol,**

**Barley House, Oakfield Grove, Bristol,** BS8 2BN, **UK**

lh14833@bristol.ac.uk

+44 (0)117 331 0128

**This material supplements, but does not replace, the peer-reviewed paper in**

***Drug and Alcohol Dependence*.**

**Supplementary Methods**

**Genotyping quality control:**

Individuals were excluded from further analysis on the basis of having incorrect gender assignments; minimal or excessive heterozygosity (0.345 for the Sanger data and 0.330 for the LabCorp data); disproportionate levels of individual missingness (>3%); evidence of cryptic relatedness (>10% IBD) and being of non-European ancestry (as detected by a multidimensional scaling analysis seeded with HapMap 2 individuals. The resulting data set consisted of 8365 individuals. After quality control, 500,527 SNPs were available for analysis.

**Genotyping imputation:**

ALSPAC genotype data generated using the Illumina HumanHap550 quad (children) and Illumina human660W quad (mothers) were first phased together using ShapeIt (version 2) and then jointly imputed to the 1000 genomes reference panel (Version 1, Phase 3, Dec 2013 Release). Genotypes were filtered to have Hardy Weinberg equilibrium p > 5 x 10^-7^ and imputation info score > 0.8. The final imputed dataset contained 8,074,398 loci.

**Supplementary Table 1:** Recoding of smoking-behaviour questions at each time-point into variables on which latent class analysis was based

| Age  Source | 13 years (N=5813)  Computer in clinic | 14 years (N=5671)  Postal questionnaire | 15 years (N=5107)  Computer in clinic | 16 years (N=4853)  Postal questionnaire | 17 years (N=3954)  Computer in clinic | 18 years (N=3209)  Postal questionnaire |
| --- | --- | --- | --- | --- | --- | --- |
| Non-smoker | “ever smoked a cigarette”= “No”  OR if “still smokes” = “No”  OR if “smoking habits in previous 6 months” = “no smoking” | “ever smoked a cigarette”= “No”  OR if “current smoking behaviour”  = “I used to smoke sometimes but I never smoke cigarettes now”  OR if “current smoking behaviour”  =”I have only ever tried smoking cigarettes once or twice” | “tried a cigarette”=”No”  OR “smoked in last 30 days” = “No” | “ever smoked a cigarette”= “No”  OR if “current smoking behaviour”  = “I used to smoke sometimes but I never smoke cigarettes now”  OR if “current smoking behaviour”  =”I have only ever tried smoking cigarettes once or twice” | “smoked a whole cigarette”=”No”  OR “smoked in last 30 days” = “No” | “smoked a whole cigarette”=”No”  OR “smoked in last 30 days” = “No” |
| Occasional smoker | “ever smoked a cigarette”= “Yes”  AND if “still smokes” = “Yes”  AND if “smoking habits in previous 6 months” = “1-3 times per month” OR “more than 4 times” | “ever smoked a cigarette”= “Yes”  AND if “current smoking behaviour”  =”I sometimes smoke cigarettes but I smoke less than one a week” | “tried a cigarette”=”Yes”  AND “smoked in last 30 days” = “Yes”  AND “smokes weekly” = “No”  AND “smokes daily”=”No” | “ever smoked a cigarette”= “Yes”  AND if “current smoking behaviour”  =”I sometimes smoke cigarettes but I smoke less than one a week” | “smoked a whole cigarette”=”Yes”  AND “smoked in last 30 days” = “Yes”  AND “smokes weekly” = “No”  AND “smokes daily”=”No” | “smoked a whole cigarette”=”Yes”  AND “smoked in last 30 days” = “Yes”  AND “smokes weekly” = “No”  AND “smokes daily”=”No” |
| Weekly smoker | “ever smoked a cigarette”= “Yes”  AND if “still smokes” = “Yes”    AND if “smoking habits in previous 6 months” = “once per week” | “ever smoked a cigarette”= “Yes”  AND if “current smoking behaviour”  =”I usually smoke between one and six cigarettes a week”  OR “I usually smoke more than six cigarettes a week, but not every day” | “tried a cigarette”=”Yes”  AND “smoked in last 30 days” = “Yes”  AND “smokes weekly” = “Yes” | “ever smoked a cigarette”= “Yes”  AND if “current smoking behaviour”  =”I usually smoke between one and six cigarettes a week”  OR “I usually smoke more than six cigarettes a week, but not every day” | “smoked a whole cigarette”=”Yes”  AND “smoked in last 30 days” = “Yes”  AND “smokes weekly” = “Yes” | “smoked a whole cigarette”=”Yes”  AND “smoked in last 30 days” = “Yes”  AND “smokes weekly” = “Yes” |
| Daily smoker | N/A ^1^ | “ever smoked a cigarette”= “Yes”  AND if “current smoking behaviour”  =”I usually smoke one or more cigarettes every day” | “tried a cigarette”=”Yes”  AND “smoked in last 30 days” = “Yes”  AND “smokes daily”=”Yes” | “ever smoked a cigarette”= “Yes”  AND if “current smoking behaviour”  =”I usually smoke one or more cigarettes every day” | “smoked a whole cigarette”=”Yes”  AND “smoked in last 30 days” = “Yes”  AND “smokes daily”=”Yes” | “smoked a whole cigarette”=”Yes”  AND “smoked in last 30 days” = “Yes”  AND “smokes daily”=”Yes” |

**Supplementary Table 2:** List of SNPs in body mass index genetic risk score

| **SNP** | **Chromosome** | **Base Pair** | **Nearest gene** | **Effect Allele** | **β** |
| --- | --- | --- | --- | --- | --- |
| rs1558902 | 16 | 52,361,075 | *FTO* | A | 0.082 |
| rs17024393 | 1 | 109,956,211 | *GNAT2* | C | 0.066 |
| rs13021737 | 2 | 622,348 | *TMEM18* | G | 0.060 |
| rs6567160 | 18 | 55,980,115 | *MC4R* | C | 0.056 |
| rs11847697 | 14 | 29,584,863 | *PRKD1* | T | 0.049 |
| rs543874 | 1 | 176,156,103 | *SEC16B* | G | 0.048 |
| rs16851483 | 3 | 142,758,126 | *RASA2* | T | 0.048 |
| rs13107325 | 4 | 103,407,732 | *SLC39A8* | T | 0.048 |
| rs1516725 | 3 | 187,306,698 | *ETV5* | C | 0.045 |
| rs2207139 | 6 | 50,953,449 | *TFAP2B* | G | 0.045 |
| rs11030104 | 11 | 27,641,093 | *BDNF* | A | 0.041 |
| rs10938397 | 4 | 44,877,284 | *GNPDA2* | G | 0.040 |
| rs12446632 | 16 | 19,842,890 | *GPRC5B* | G | 0.040 |
| rs7899106 | 10 | 87,400,884 | *GRID1* | G | 0.040 |
| rs2287019 | 19 | 50,894,012 | *QPCTL* | C | 0.036 |
| rs11727676 | 4 | 145,878,514 | *HHIP* | T | 0.036 |
| rs12429545 | 13 | 53,000,207 | *OLFM4* | A | 0.033 |
| rs3101336 | 1 | 72,523,773 | *NEGR1* | C | 0.033 |
| rs7138803 | 12 | 48,533,735 | *BCDIN3D* | A | 0.032 |
| rs3888190 | 16 | 28,796,987 | *ATP2A1* | A | 0.031 |
| rs11191560 | 10 | 104,859,028 | *NT5C2* | C | 0.031 |
| rs11057405 | 12 | 121,347,850 | *CLIP1* | G | 0.031 |
| rs17001654 | 4 | 77,348,592 | *SCARB2* | G | 0.031 |
| rs10182181 | 2 | 25,003,800 | *ADCY3* | G | 0.031 |
| rs16951275 | 15 | 65,864,222 | *MAP2K5* | T | 0.031 |
| rs12016871 | 13 | 26,915,782 | *MTIF3* | T | 0.030 |
| rs13078960 | 3 | 85,890,280 | *CADM2* | G | 0.030 |
| rs3810291 | 19 | 52,260,843 | *ZC3H4* | A | 0.028 |
| rs13191362 | 6 | 162,953,340 | *PARK2* | A | 0.028 |
| rs3817334 | 11 | 47,607,569 | *MTCH2* | T | 0.026 |
| rs2112347 | 5 | 75,050,998 | *POC5* | T | 0.026 |
| rs2075650 | 19 | 50,087,459 | *TOMM40* | A | 0.026 |
| rs2121279 | 2 | 142,759,755 | *LRP1B* | T | 0.025 |
| rs10968576 | 9 | 28,404,339 | *LINGO2* | G | 0.025 |
| rs17094222 | 10 | 102,385,430 | *HIF1AN* | C | 0.025 |
| rs12566985 | 1 | 74,774,781 | *FPGT-TNNI3K* | G | 0.024 |
| rs7141420 | 14 | 78,969,207 | *NRXN3* | T | 0.024 |
| rs1016287 | 2 | 59,159,129 | *LINC01122* | T | 0.023 |
| rs758747 | 16 | 3,567,359 | *NLRC3* | T | 0.023 |
| rs10132280 | 14 | 24,998,019 | *STXBP6* | C | 0.023 |
| rs657452 | 1 | 49,362,434 | *AGBL4* | A | 0.023 |
| rs7903146 | 10 | 114,748,339 | *TCF7L2* | C | 0.023 |
| rs12286929 | 11 | 114,527,614 | *CADM1* | G | 0.022 |
| rs17405819 | 8 | 76,969,139 | *HNF4G* | T | 0.022 |
| rs7243357 | 18 | 55,034,299 | *GRP* | T | 0.022 |
| rs7599312 | 2 | 213,121,476 | *ERBB4* | G | 0.022 |
| rs11165643 | 1 | 96,696,685 | *PTBP2* | T | 0.022 |
| rs205262 | 6 | 34,671,142 | *C6orf106* | G | 0.022 |
| rs2650492 | 16 | 28,240,912 | *SBK1* | A | 0.021 |
| rs4256980 | 11 | 8,630,515 | *TRIM66* | G | 0.021 |
| rs12885454 | 14 | 28,806,589 | *PRKD1* | C | 0.021 |
| rs12401738 | 1 | 78,219,349 | *FUBP1* | A | 0.021 |
| rs11126666 | 2 | 26,782,315 | *KCNK3* | A | 0.021 |
| rs1167827 | 7 | 75,001,105 | *HIP1* | G | 0.020 |
| rs2820292 | 1 | 200,050,910 | *NAV1* | C | 0.020 |
| rs2365389 | 3 | 61,211,502 | *FHIT* | C | 0.020 |
| rs2176598 | 11 | 43,820,854 | *HSD17B12* | T | 0.020 |
| rs2033732 | 8 | 85,242,264 | *RALYL* | C | 0.019 |
| rs2033529 | 6 | 40,456,631 | *TDRG1* | G | 0.019 |
| rs17724992 | 19 | 18,315,825 | *PGPEP1* | A | 0.019 |
| rs9400239 | 6 | 109,084,356 | *FOXO3* | C | 0.019 |
| rs3849570 | 3 | 81,874,802 | *GBE1* | A | 0.019 |
| rs9925964 | 16 | 31,037,396 | *KAT8* | A | 0.019 |
| rs1928295 | 9 | 119,418,304 | *TLR4* | T | 0.019 |
| rs6804842 | 3 | 25,081,441 | *RARB* | G | 0.019 |
| rs1000940 | 17 | 5,223,976 | *RABEP1* | G | 0.019 |
| rs4740619 | 9 | 15,624,326 | *C9orf93* | T | 0.018 |
| rs29941 | 19 | 39,001,372 | *KCTD15* | G | 0.018 |
| rs12940622 | 17 | 76,230,166 | *RPTOR* | G | 0.018 |
| rs11583200 | 1 | 50,332,407 | *ELAVL4* | C | 0.018 |
| rs3736485 | 15 | 49,535,902 | *DMXL2* | A | 0.018 |
| rs1528435 | 2 | 181,259,207 | *UBE2E3* | T | 0.018 |
| rs11688816 | 2 | 62,906,552 | *EHBP1* | G | 0.017 |
| rs6477694 | 9 | 110,972,163 | *EPB41L4B* | C | 0.017 |
| rs10733682 | 9 | 128,500,735 | *LMX1B* | A | 0.017 |
| rs1808579 | 18 | 19,358,886 | *C18orf8* | C | 0.017 |

Note: SNP positions reported according to Build 36. Betas, effect alleles (positive strand) and nearest gene taken from GIANT publications (Speliotes 2010), (Locke 2015)

**Supplementary Table 3:** Fit statistics for models assuming different numbers of classes for the longitudinal latent class analysis

| Fit statistic | Number of classes | | | | |
| --- | --- | --- | --- | --- | --- |
|  | 2 | 3 | 4 | 5 | 6 |
| BIC | 22136.2 | 21541.9 | 21469.2 | 21528.5 | 21596.8 |
| Entropy | 0.867 | 0.799 | 0.818 | 0.823 | 0.806 |
| BLRT P-value | <0.0001 | <0.0001 | <0.0001 | <0.0001 | <0.0001 |
| VLMR P-value | <0.0001 | <0.0001 | 0.0010 | 1.0000 | 1.0000 |
| LMR P-value | <0.0001 | <0.0001 | 0.0010 | 1.0000 | 1.0000 |
| Smallest class size | 19.56% | 7.20% | 2.89% | 2.07% | 1.52% |

BIC - Bayesian Information Criterion; BLRT - bootstrapped likelihood ratio test for k compared with k-1 classes; LMR- Lo-Mendell-Rubin likelihood ratio test for k compared with k-1 classes; VLMR – Vuong-Lo-Mendell-Rubin likelihood ratio test for k compared with k-1 classes. A 4 class model was chosen based on the following criteria: reasonable fit to the data (low BIC); good separation of the classes (high entropy); and a sufficient number of observations in smallest class (n = 154), better fit than next lowest number of classes (P < 0.001 from all three different likelihood ratio tests of 4 compared with 3 classes) and the LMRT and VLMRT indicated that there was no gain in adding another class (P > 0.05).

**Supplementary Table 4:** Characteristics of individuals included in the analysis

|  |  | Observational Data-set (n=3754) | | MR Data-set (n=4017) | |
| --- | --- | --- | --- | --- | --- |
|  |  | Males (n=1732) | Females (n=2022) | Males (n=1768) | Females (n=2249) |
| BMI (kg/m^2^) | Mean (SD) | 17.9 (2.8) | 18.2 (3.0) | 17.9 (2.8) | 18.2 (3.0) |
| Body Dissatisfaction: N (%) | Body Satisfaction  Body Dissatisfaction | 1119 (72.7)  421 (27.3) | 1183 (65.4)  626 (34.6) | 938 (72.4)  358 (27.6) | 1067 (66.6)  536 (33.4) |
| Age at BMI assessment (years) | Mean (SD) | 10.6 (0.2) | 10.6 (0.2) | 10.6 (0.20) | 10.6 (0.22) |
| Maternal Education: N (%) | CSE  Vocational  O Level  A Level  Degree | 107 (6.2)  130 (7.5)  586 (33.8)  532 (30.7)  377 (21.8) | 159 (7.9)  136 (6.7)  708 (35.0)  594 (29.4)  425 (21.0) | 120 (7.1)  129 (7.6)  583 (34.4)  494 (29.2)  368 (21.7) | 183 (8.5)  145 (6.8)  758 (35.4)  613 (28.6)  443 (20.7) |
| Maternal Smoking: N (%) | Current Smoker  Former Smoker  Never Smoked | 209 (12.1)  464 (26.8)  1059 (61.1) | 276 (13.7)  508 (25.1)  1238 (61.2) | 206 (12.9)  442 (27.7)  949 (59.4) | 271 (13.8)  510 (26.0)  1178 (60.1) |
| Housing Tenure: N (%) | Mortgaged  Owned  Council Rented  Rented Private  Other | 1499 (86.6)  35 (2.0)  75 (4.3)  95 (5.5)  28 (1.6) | 1713 (84.7)  43 (2.1)  108 (5.3)  108 (5.3)  50 (2.5) | 1496 (86.1)  36 (2.1)  83 (4.8)  88 (5.1)  35 (2.0) | 1826 (82.8)  47 (2.1)  148 (6.7)  101 (4.6)  84 (3.8) |
| Crowding Status: N (%) | pprm ≤0.5  0.5 < pprm ≤ 0.75  0.75 < pprm ≤ 1  1< pprm | 963 (55.6)  534 (30.8)  194 (11.2)  41 (2.4) | 1072 (53.0)  628 (31.1)  275 (13.6)  47 (2.3) | 955 (55.5)  514 (29.9)  206 (12.0)  45 (2.6) | 1133 (51.8)  662 (30.3)  327 (15.0)  64 (2.9) |
| Parity (Number of Pregnancies) | Mean (SD) | 0.704 (0.87) | 0.708 (0.84) | 0.709 (0.89) | 0.722 (0.82) |

BMI: body mass index; CSE: certificate of secondary education (Exams at age 16); Vocational: Education specific to a trade (e.g. plumbing, electrician), O Level: Exams at age 16 aimed at more academic students; A Level: School leaving exams at age 18; Degree: University degree; pprm: persons per room.

**Supplementary Table 5.** Association of one unit increase in body mass index on covariates and body dissatisfaction

| Variable | BMI (kg/m^2^) | | | | | |
| --- | --- | --- | --- | --- | --- | --- |
|  | Males (N=1732) | | | Females (N=2022) | | |
|  | Odds ratio | 95% C.I. | P-Value | Coefficient | 95% C.I. | P-Value |
| Parity^1^ | 0.96 | (0.87, 1.06) | 0.410 | 1.01 | (0.93, 1.09) | 0.830 |
| Mothers Education ^2^ | 1.04 | (1.01, 1.08) | 0.018 | 1.05 | (1.02, 1.08) | 0.001 |
| Maternal Smoking ^3^ | 1.03 | (0.99, 1.06) | 0.106 | 1.04 | (1.01, 1.07) | 0.014 |
| Housing  Tenure ^4^ | 1.02 | (0.97, 1.07) | 0.482 | 1.05 | (1.00, 1.09) | 0.030 |
| Crowding  Status ^5^ | 1.01 | (0.98, 1.04) | 0.578 | 1.02 | (0.99, 1.05) | 0.233 |
| Body  Dissatisfaction^6^ | 1.23 | (1.18, 1.28) | <0.001 | 1.31 | (1.26, 1.36) | <0.001 |

^1^ Parity (≤2 Pregnancies=0 3≤ Pregnancies=1); ^2^ Mothers Education (A Level/Degree=0 CSE/Vocational/O Level=1); ^3^ Maternal Smoking (Never=0 Ever=1); ^4^ Housing Tenure (Owned/Mortgaged=0 Other=1); ^5^ Crowding Status (‘<= 0.5’=0 ‘0.5<’=1); ^6^ Body Dissatisfaction at age 10.5 (None=0 Some=1).

**Supplementary Table 6.** Effect of one standard deviation increase in risk score on covariates and observed body mass index / body dissatisfaction

| Variable | Males (n=1768) | | | Females (n=2249) | | |
| --- | --- | --- | --- | --- | --- | --- |
|  | Coefficient^1^ | 95% C.I. | P Value | Coefficient^1^ | 95% C.I. | P Value |
| Parity^2^ | 0.99 | (0.88, 1.12) | 0.86 | 1.05 | (0.94, 1.17) | 0.420 |
| Mothers Education ^3^ | 0.99 | (0.90, 1.09) | 0.89 | 1.01 | (0.93, 1.10) | 0.80 |
| Maternal Smoking ^4^ | 1.05 | (0.95, 1.16) | 0.32 | 0.98 | (0.90, 1.08) | 0.68 |
| Housing Tenure ^5^ | 0.91 | (0.78, 1.05) | 0.18 | 1.04 | (0.93, 1.17) | 0.49 |
| Crowding Status ^6^ | 1.01 | (0.92, 1.11) | 0.88 | 1.03 | (0.95, 1.13) | 0.43 |
| Body Dissatisfaction^7^ | 1.08 | (0.95, 1.22) | 0.24 | 1.11 | (1.00, 1.23) | 0.056 |
| BMI at Age 10.5 years ^8^ | 0.74 | (0.57, 0.90) | <0.001 | 0.65 | (0.51, 0.79) | < 0.001 |

Results are shown per 1 standard deviation increase in genetic risk score. ^1^ Logistic Regression (Odds ratios) for binary variable and Linear Regression (Beta coefficients) for continuous variables; ^2^ Parity (≤2 Pregnancies=0 3≤ Pregnancies=1); ^3^ Mothers Education (A Level/Degree=0 CSE/Vocational/O Level=1); ^4^ Maternal Smoking (Never=0 Ever=1); ^5^ Housing Tenure (Owned/Mortgaged=0 Other=1); ^6^ Crowding Status (‘<= 0.5’=0 ‘0.5<’=1); ^7^ Body Dissatisfaction at Age 10.5 (None=0 Some=1); ^8^ BMI at Age 10.5 (kg/m^2^).

**Supplementary Table 7.** Extension of Table 4 with a pleiotropic SNP (rs11030104 in *BDNF*) removed

|  | Class (percentage membership) | | | | |
| --- | --- | --- | --- | --- | --- |
|  | Never  (70.1%) | Experimenters  (17.3%) | Late Onset  (9.4%) | Early Onset  (3.2%) | P value |
| Males  (n=1768) | 1.00 (REF) | 0.90 (0.70, 1.09) | 0.94 (0.76, 1.12) | 1.00 (0.65, 1.34) | 0.64 |
| Females  (n=2249) | 1.00 (REF) | 1.09 (0.94, 1.23) | 0.94 (0.72, 1.16) | 1.20 (0.92, 1.48) | 0.47 |
| Total  (n=4017) | 1.00 (REF) | 1.01 (0.90, 1.13) | 0.94 (0.80, 1.08) | 1.13 (0.91, 1.34) | 0.67 |

**Supplementary Table 8:** Triangulation to estimate expected association between body mass index polygenic risk score and smoking initiation compared with the observed association

|  |  | *Experimenters* | *Late-onset* | *Early-Onset* |
| --- | --- | --- | --- | --- |
| **Odds ratio for BMI on smoking initiation (fully adjusted)** | *Males* | 0.93 (0.84, 1.02) | 0.99 (0.91, 1.06) | 1.04 (0.95, 1.13) |
|  | *Females* | 1.04 (0.99, 1.10) | 1.01 (0.94, 1.09) | 1.11 (1.04, 1.18) |
| **Effect of one S.D. increase in polygenic risk score on observed BMI (kg/m^2^)** | *Males* | 0.74 (0.57, 0.90)_1_ | | |
|  | *Females* | 0.65 (0.51, 0.79)_1_ | | |
| **Expected O.R. for polygenic risk score on smoking initiation** | *Males* | 0.96 | 0.99 | 1.03 |
|  | *Females* | 1.03 | 1.01 | 1.08 |
| **Observed O.R. for polygenic risk score on smoking initiation** | *Males* | 0.90 (0.70, 1.11) | 0.94 (0.75, 1.12) | 1.00 (0.62, 1.37) |
|  | *Females* | 1.09 (0.94, 1.23) | 0.96 (0.74, 1.18) | 1.21 (0.93, 1.48) |

^1^ Effect of polygenic risk score on observed BMI calculated in the whole sample to increase power

**Supplementary Figure Legends**

**Supplementary Figure 1:** Flowcharts showing how the final three samples (observational BMI, the body dissatisfaction subset and the MR sample) were reached from an original population of 15,455

**Supplementary Figure 1:** Flowcharts showing how the final three samples (observational BMI, the body dissatisfaction subset and the MR sample) were reached from an original population of 15,455


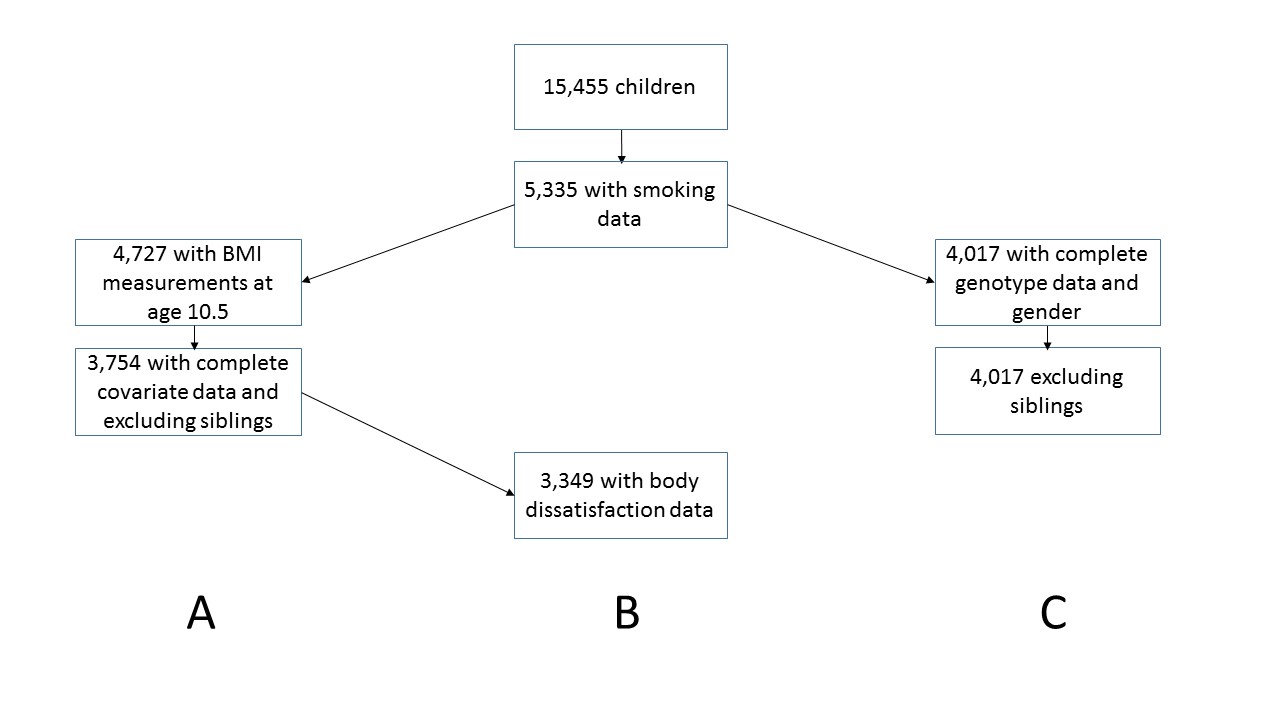


A – Body mass index (BMI) and smoking

B – Body dissatisfaction and smoking

C – BMI genetic risk score and smoking
